# Heterotrophic nitrification by *Alcaligenes faecalis* links organic and inorganic nitrogen metabolism

**DOI:** 10.1093/ismejo/wrae174

**Published:** 2024-09-10

**Authors:** Ya-Ling Qin, Zong-Lin Liang, Guo-Min Ai, Wei-Feng Liu, Yong Tao, Cheng-Ying Jiang, Shuang-Jiang Liu, De-Feng Li

**Affiliations:** State Key Laboratory of Microbial Resources, Institute of Microbiology, Chinese Academy of Sciences, No. 1 Beichen West Road, Chaoyang District, Beijing 100101, P. R. China; School of Life Sciences, University of Chinese Academy of Sciences, No. 1 Yanqihu East Road, Huairou District, Beijing 100049, P. R. China; State Key Laboratory of Microbial Resources, Institute of Microbiology, Chinese Academy of Sciences, No. 1 Beichen West Road, Chaoyang District, Beijing 100101, P. R. China; School of Life Sciences, University of Chinese Academy of Sciences, No. 1 Yanqihu East Road, Huairou District, Beijing 100049, P. R. China; State Key Laboratory of Microbial Resources, Institute of Microbiology, Chinese Academy of Sciences, No. 1 Beichen West Road, Chaoyang District, Beijing 100101, P. R. China; State Key Laboratory of Microbial Resources, Institute of Microbiology, Chinese Academy of Sciences, No. 1 Beichen West Road, Chaoyang District, Beijing 100101, P. R. China; State Key Laboratory of Microbial Resources, Institute of Microbiology, Chinese Academy of Sciences, No. 1 Beichen West Road, Chaoyang District, Beijing 100101, P. R. China; School of Life Sciences, University of Chinese Academy of Sciences, No. 1 Yanqihu East Road, Huairou District, Beijing 100049, P. R. China; State Key Laboratory of Microbial Resources, Institute of Microbiology, Chinese Academy of Sciences, No. 1 Beichen West Road, Chaoyang District, Beijing 100101, P. R. China; School of Life Sciences, University of Chinese Academy of Sciences, No. 1 Yanqihu East Road, Huairou District, Beijing 100049, P. R. China; State Key Laboratory of Microbial Resources, Institute of Microbiology, Chinese Academy of Sciences, No. 1 Beichen West Road, Chaoyang District, Beijing 100101, P. R. China; School of Life Sciences, University of Chinese Academy of Sciences, No. 1 Yanqihu East Road, Huairou District, Beijing 100049, P. R. China; State Key Laboratory of Microbial Resources, Institute of Microbiology, Chinese Academy of Sciences, No. 1 Beichen West Road, Chaoyang District, Beijing 100101, P. R. China; School of Life Sciences, University of Chinese Academy of Sciences, No. 1 Yanqihu East Road, Huairou District, Beijing 100049, P. R. China

**Keywords:** Heterotrophic nitrification, Alcaligenes faecalis, Heterotrophic ammonia oxidation, dirammox, dnfABC

## Abstract

Heterotrophic nitrification remains a mystery for decades. It has been commonly hypothesized that heterotrophic nitrifiers oxidize ammonia to hydroxylamine and then to nitrite in a way similar to autotrophic AOA and AOB. Recently, heterotrophic nitrifiers from *Alcaligenes* were found to oxidize ammonia to hydroxylamine and then to N_2_ (“dirammox”, direct ammonia oxidation) by the gene cluster *dnfABC* with a yet-to-be-reported mechanism*.* The role of a potential glutamine amidotransferase DnfC clues the heterotrophic ammonia oxidation might involving in glutamine. Here, we found *Alcaligenes faecalis* JQ135 could oxidize amino acids besides ammonia. We discovered that glutamine is an intermediate of the dirammox pathway and the glutamine synthetase gene *glnA* is essential for both *A. faecalis* JQ135 and the *Escherichia coli* cells harboring *dnfABC* gene cluster to oxidize amino acids and ammonia. Our study expands understanding of heterotrophic nitrifiers and challenges the classical paradigm of heterotrophic nitrification.

## Introduction

Microbial ammonia oxidation plays a key role in the biogeochemical nitrogen cycle. Two ammonia oxidation pathways in chemolithotrophs have been extensively studied, i.e., nitrification that is usually referred as the oxidation of ammonia to nitrite and nitrate via the intermediate hydroxylamine, and anammox that is referred as the oxidation of ammonia using nitrite as the electron acceptor to generate dinitrogen gas [1–4]. Heterotrophic nitrification, the oxidation of ammonia and any other reduced nitrogenous compounds in heterotrophs has been discovered for over a century, occurs widely across *Proteobacteria*, *Bacteroidetes*, *Firmicutes*, *Actinobacteria*, and fungi, and yet remains a mystery in the nitrogen cycle [5–10]. Most heterotrophic nitrifiers were observed to produce hydroxylamine, nitrite, and/or nitrate, mirroring the nitrification process in ammonia-oxidizing archaea and bacteria (AOA and AOB), but they cannot obtain energy to fix carbon dioxide from this process as AOA and AOB can [11–16]. Although the accumulations of nitrite/nitrate were limited, the productions of N_2_ and N_2_O were confirmed. Additionally, it was discovered that the majority of heterotrophic nitrifiers possess the complete set of enzymes necessary for a denitrification pathway, enabling the conversion of nitrite or nitrate to N_2_. Therefore, it was proposed that heterotrophic nitrifiers first oxidized ammonia to nitrite/nitrate via the heterotrophic nitrification pathway and then reduced nitrite/nitrate to gas products under aerobic conditions [14, 17]. Productions of hydroxylamine and nitrite were also observed when some heterotrophic nitrifiers were cultured with organic nitrogenous compounds [12, 18–24]. These differences indeed suggested that the oxidation of ammonia in heterotrophs and autotrophs may recruit different enzymes, intermediates, pathways, and mechanisms.

*Alcaligenes* has attracted significant attention in wastewater treatment for its superior nitrogen removal efficiency compared to autotrophs. It is known for its highly active heterotrophic nitrification, capable of oxidizing ammonia, hydroxylamine, and pyruvic oxime, and possesses all the enzymes required for denitrification, despite not encoding known AMO and HAO enzymes [7, 16, 23, 25–32]. Recently, *Alcaligenes* species, including *Alcaligenes ammonioxydans* HO-1 and *A. faecalis* JQ135, were found to perform direct ammonia oxidation (“dirammox”) which contributed to almost all of the N_2_ production (about half of the initial ammonium amounts) of *Alcaligenes* cells under aerobic conditions. This process involves oxidization of ammonia to hydroxylamine and then directly to N_2_ via a gene cluster *dnfABC* in the presence of organic carbon sources. The enzyme DnfA was predicted as a di-iron oxidase, related to ammonia oxidation, and also identified as the oxidase responsible for oxidizing hydroxylamine to N_2_ with the assistance of an electron shuttle protein DnfB [32–34]. Actually, the DnfA homologs, CmlI and AurF, could oxidize the amino group of amines to the nitro group via their diiron centers [35, 36], agreeing with the potential dual role of DnfA in ammonia (or amine) oxidation and hydroxylamine oxidation. DnfC was predicted as a potential glutamine amidotransferase. Miao and colleagues suggested that DnfABC oxidized glutamine to dinitrogen gas in vitro, though lacking direct biochemical evidence to support the oxidation of glutamine by DnfA [37]. A recent study also proposed that *A. faecalis* produced hydroxylamine from an unidentified organic intermediate [38]. Nevertheless, those findings clued that heterotrophic nitrification by *Alcaligenes* might recruit a different pathway from those in AOA and AOB.

Here, we investigated whether the natural heterotrophic nitrifier *A. faecalis* JQ135 and the engineered heterotrophic nitrifier *E. coli* cells harboring *dnfABC* could oxidize amino acids through the dirammox pathway and identified the possible common intermediate in the ammonia and amine oxidation by the dirammox pathway. Our findings reveal that heterotrophic nitrifier *A. faecalis* JQ135 can oxidize ammonia and amines using glutamine as a shared intermediate. This discovery challenges the traditional paradigm regarding heterotrophic nitrification.

## Materials and methods

### Strains, plasmids, media, and cultivation

All bacterial strains and plasmids used in this study are listed in Supplementary Table 1. The construction of plasmid pBAD-*dnfABC,* preparation of media and cultivation of *A. faecalis* JQ135 and *E. coli* cells followed the established protocols as previously described [32, 33]. The HNM media contains (per liter) 0.66 g (NH_4_)_2_SO_4_, 4.72 g sodium succinate, 0.50 g KH_2_PO_4_, 1.25 g Na_2_HPO_4_·12H_2_O, 0.20 g MgSO_4_·7H_2_O, 2.00 ml trace element solution at pH 7.5–8.0. The trace element solution contains (per liter) 57.10 g EDTA·2Na, 3.90 g ZnSO_4_·7H_2_O, 7.00 g CaCl_2_·2H_2_O, 1.00 g MnCl_2_·4H_2_O, 5.00 g FeSO_4_·7H_2_O, 1.10 g (NH_4_)_6_Mo_7_O_24_·4H_2_O, 1.60 g CuSO_4_·5H_2_O, 1.60 g CoCl_2_·6H_2_O at pH 6.0. In the modified HNM media, 10 mM of various amino acids were utilized as the nitrogen source instead of ammonia.

### Oxidation of organic nitrogen sources

For growth-dependent assays, *A. faecalis* JQ135 was cultivated in HNM medium or modified HNM medium. The flasks were incubated at 30°C with a shaking speed of 160 rpm and an inoculation amount of 1% (v/v). Samples were harvested periodically to measure OD_600_ and the accumulation of hydroxylamine.

For the whole cell transformation (growth-independent) assays, JQ135 and JQ135Δ*glnA*::*glnA* cells were cultured overnight with HNM media, harvested, and transferred into the media containing different nitrogen sources with a final OD_600_ of 1. JQ135Δ*glnA* cells were cultured with HNM where ammonia was replaced by 10 mM glutamine and subjected on the assays. *E. coli* and variant cells harboring pBAD-*dnfABC* were cultured in LB medium, induced by arabinose, harvested, and subsequently transferred into the media with a final OD_600_ of 1, as previously described [32].

### Gene deletion and complementation

The genetic deletion mutant JQ135Δ*glnA* and the complementary strain JQ135Δ*glnA*::*glnA_Afe_* were constructed via a two-step homologous recombination method as previously described [33]. The single gene knockout *E. coli* mutants were derived from *E. coli* BW25113 strain, as a part of Keio collection obtained from the National Institute of Genetics, Japan [39]. The complementary strain *E.coli*Δ*glnA*::*glnA_Eco_* was constructed as described in JQ135. All primers used in this study are listed in Supplementary Table 2.

### Analytical methods

Bacterial growth was monitored by spectrophotometrically measured at 600 nm. Quantitative detections of hydroxylamine were measured by the method as previously described [32, 40]. The expression of DnfA/DnfC in JQ135 was assessed by western blotting when the yield of hydroxylamine reached its highest or the cells entered the logarithmic phase. The sample cells used for western blotting were harvest and their concentrations were adjusted to OD_600_ of 1.0 before lysis. ^15^N_2_ was determined by GC–MS as previously described [32].

## Results

### Amino acids could be oxidized to hydroxylamine by *A. faecalis* JQ135

**Figure 1:**
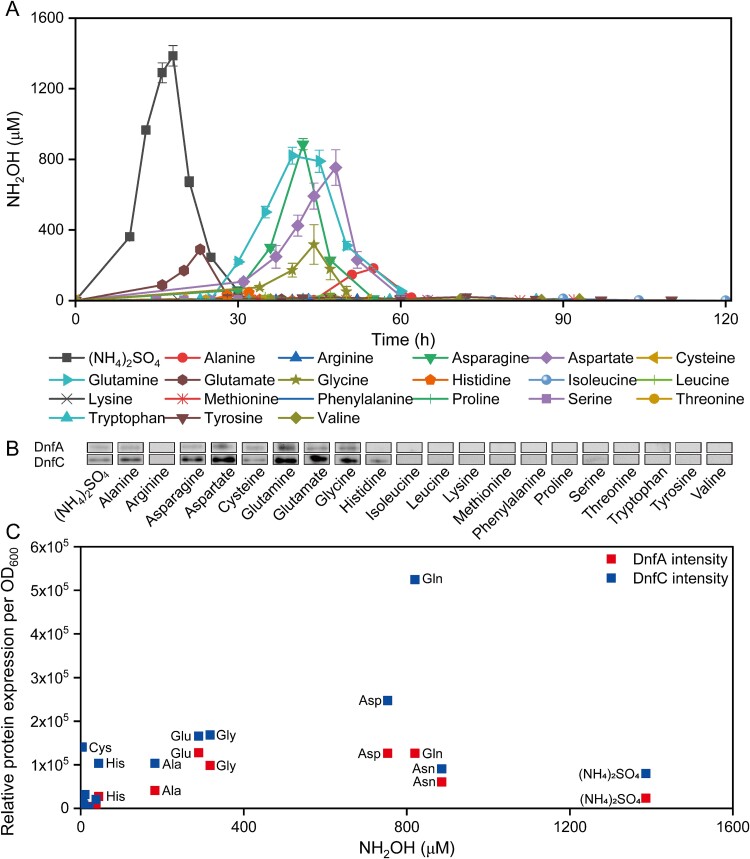
***A. faecalis* JQ135 cells oxidize amino acids besides ammonia in growth-dependent assays.** (A, B) hydroxylamine accumulation curves (A) and expressions of DnfA/DnfC (B) in JQ135 cells cultured with ammonia or amino acids. (C) the plot between the hydroxylamine accumulation and the relative expression levels of DnfA/DnfC under various nitrogen sources. The data are represented as the mean ± s.d. of biological triplicates.

To investigate the potential nitrogen sources that could be oxidized to hydroxylamine by the dirammox pathway, *A. faecalis* JQ135 was cultured with modified HNM media where the nitrogen source (ammonia) was substituted with 20 different amino acids. JQ135 cells exhibited growth in these media, reaching a maximum OD_600_ of ~0.8–1.2 within 26–100 h (Fig. 1A, Supplementary Fig. 1). Significant hydroxylamine accumulations, with a maximum of ~800 μM, were observed in media containing asparagine (885.8 ± 31.8 μM at 42 h), aspartate (753.3 ± 100.5 μM at 48 h), or glutamine (820.4 ± 48.2 μM at 40 h), respectively, which were comparable to that in medium containing ammonia (1386.7 ± 58.6 μM at 18 h) (Fig. 1A, Supplementary Fig. 1). Moderate level of hydroxylamine accumulations, ~200–300 μM, were observed in media containing alanine (182.5 ± 5.4 μM at 55 h), glutamate (289.6 ± 10.9 μM at 23 h) or glycine (317.5 ± 112.0 μM at 44 h). Limited hydroxylamine accumulations were observed in media containing histidine (44.6 ± 11.3 μM at 32 h) or leucine (37.9 ± 3.6 μM at 47 h) (Fig. 1A, Supplementary Fig. 1). No significant hydroxylamine accumulation was observed in media containing other amino acids (Fig. 1A, Supplementary Fig. 1). Since hydroxylamine is only an intermediate, no hydroxylamine accumulation for those amino acids did not exclude the possibility that JQ135 oxidized those amino acids to hydroxylamine and then quickly to N_2_. Further experimental evidence demonstrated that the deletion of *dnfA* abolished the ability to produce hydroxylamine when utilizing different amino acids (Supplementary Fig. 2A). Therefore, we conclude that JQ135 oxidizes amino acids to hydroxylamine through the dirammox pathway.

The expression levels of DnfA/DnfC in different samples were assayed by western blotting, with bacterial samples collected at the point of maximal hydroxylamine accumulation for those amino acids that yielded hydroxylamine or at the time when the maximum OD_600_ was reached for those amino acids that did not yield hydroxylamine (Fig. 1B). Cells cultured with alanine, asparagine, aspartate, glutamine, glutamate, or glycine exhibited comparable or higher expression levels of DnfA/DnfC compared to cells cultured with ammonia, indicating significant hydroxylamine accumulation associated with these amino acids. In contrast, cells cultured with histidine displayed similar expression levels of DnfA/DnfC as those with ammonia despite limited hydroxylamine accumulation. Cells cultured with cysteine or serine exhibited significant expressions of DnfA and/or DnfC despite not producing hydroxylamine. The other amino acids neither induced the expression of DnfA/DnfC nor were oxidized to hydroxylamine (Fig. 1B). These findings indicate that the expression of DnfA/DnfC is a prerequisite for hydroxylamine accumulation. However, a positive correlation between hydroxylamine accumulation and the expression of DnfA/DnfC was not observed (Fig. 1C), indicating that the production of hydroxylamine is likely influenced by additional factors beyond the expression of *dnfABC*.

### In the whole cell transformation assays, *A. faecalis* JQ135 and *E. Coli* harboring *dnfABC* oxidized amino acids to hydroxylamine

**Figure 2:**
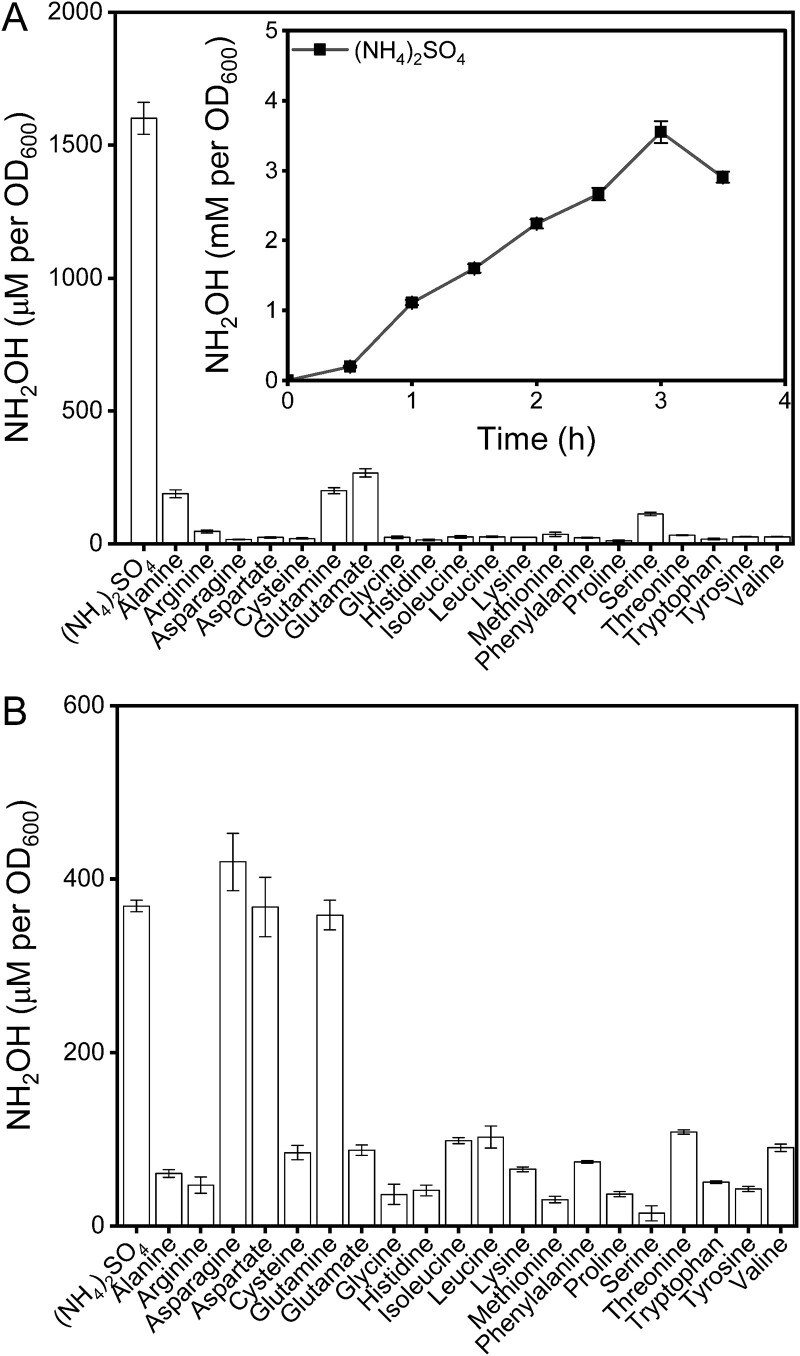
***A. faecalis* JQ135 and *E. Coli* cells harboring *dnfABC* oxidize amino acids besides ammonia in whole cell transformation assays.** (A, B) the accumulations of hydroxylamine in JQ135 cells (A) and *E. Coli* cells harboring *dnfABC* (B) cultured with various nitrogen sources at 90 min. The insert panel shows hydroxylamine accumulation curve of JQ135 cells cultured with ammonia. The data are represented as the mean ± s.d. of biological triplicates.

To mitigate the potential impact of varying DnfA/DnfC expression levels, whole cell transformation assays were conducted to directly assess the oxidation of amino acids to hydroxylamine. JQ135 cells cultured overnight were collected and then transferred into media containing either ammonia or various amino acids, with a final cell concentration adjusted to an OD_600_ of 1. Cells incubated with ammonia produced hydroxylamine with a linear accumulation rate of 20.6 ± 1.4 μM min^−1^, reaching a maximum hydroxylamine concentration of 3550 μM ± 157.3 μM at 180 min (Fig. 2A). The observed linear rate in hydroxylamine accumulation over a 180- min period indicates that the expression of DnfA/DnfC in resting cells is not significantly regulated by the presence of ammonia within a short timeframe. Substantial hydroxylamine accumulations were observed in media containing alanine (188.8 ± 14.5 μM), glutamine (200.0 ± 11.3 μM), glutamate (266.3 ± 15.2 μM) or serine (111.3 ± 6.5 μM), whereas limited hydroxylamine accumulations (11.25 ± 4.0 μM – 46.25 ± 5.4 μM) were observed with other amino acids at 90 min (Fig. 2A, Supplementary Fig. 3A). The hydroxylamine accumulation was substantially lower than that for ammonia (1601.3 ± 60.5 μM at 90 min), indicating a preference of JQ135 for oxidizing ammonia over amino acids (Fig. 2A, Supplementary Fig. 3A). Further experimental evidence demonstrated that cells with a deletion of *dnfA* did not produce hydroxylamine when incubated with ammonia or various amino acids, further supporting the idea that JQ135 recruits DnfABC to oxidize amino acids (Supplementary Fig. 2B).

To confirm the potential nitrogen sources for the dirammox pathway, an artificial nitrifier, engineered *E. coli* cells harboring *dnfABC*, was used in whole cell transformation assays. Cells cultured and induced in LB medium were collected and then transferred into media containing ammonia or various amino acids, with a final cell concentration adjusted to an OD_600_ of 1. The *E. coli* cells harboring *dnfABC* efficiently oxidized both ammonia and amino acids to hydroxylamine at 90 min (Fig. 2B). Significant hydroxylamine accumulations were observed in media containing asparagine (419.9 ± 33.2 μM), aspartate (367.9 ± 34.4 μM), or glutamine (358.6 ± 17.2 μM), respectively, which were comparable to that in medium containing ammonia (369.0 ± 6.7 μM) and significantly higher than those in media containing the other amino acids (14.8 ± 8.7 μM – 108.4 ± 2.3 μM) (Supplementary Fig. 3B). These findings indicate that the engineered heterotrophic nitrifier *E. coli* cells are capable of oxidizing ammonia and amino acids to hydroxylamine via DnfABC.

### Gene *glnA* is the only one essential for ammonia and amino acid oxidation among those well-studied nitrogen metabolism genes

In the above assays, it was observed that JQ135 and *E. coli* cells harboring *dnfABC* were capable of oxidizing ammonia, glutamine, and some other amino acids, resulting in a significant accumulation of hydroxylamine. This implies the possibility for interconversion between these compounds, where some may transform into one another before undergoing oxidation to produce hydroxylamine. The nitrogen metabolism in *E. coli* has been extensively studied and the enzymes involved in the transformation of ammonia, asparagine, aspartate, and glutamine have been investigated, including aspartate transaminase AspC, asparaginase amidohydrolase IaaA, AnsA and AnsB, aspartate ammonia ligase AsnA and AsnB, glutamate dehydrogenase (GDH) GdhA, glutamine synthetase GlnA, glutaminase GlsA and GlsB, and glutamate synthase (glutamine-oxoglutarate amidotransferase [GOGAT]) GltBD (Supplementary Fig. 4A). To explore the potential heterotrophic nitrification pathway and the roles of these genes in the ammonia and amine oxidation, *E. coli* cells with individual gene deletion were transformed with the plasmid carrying *dnfABC* and subjected to hydroxylamine accumulation assays using ammonia, asparagine, aspartate, or glutamine as substrates.

**Figure 3:**
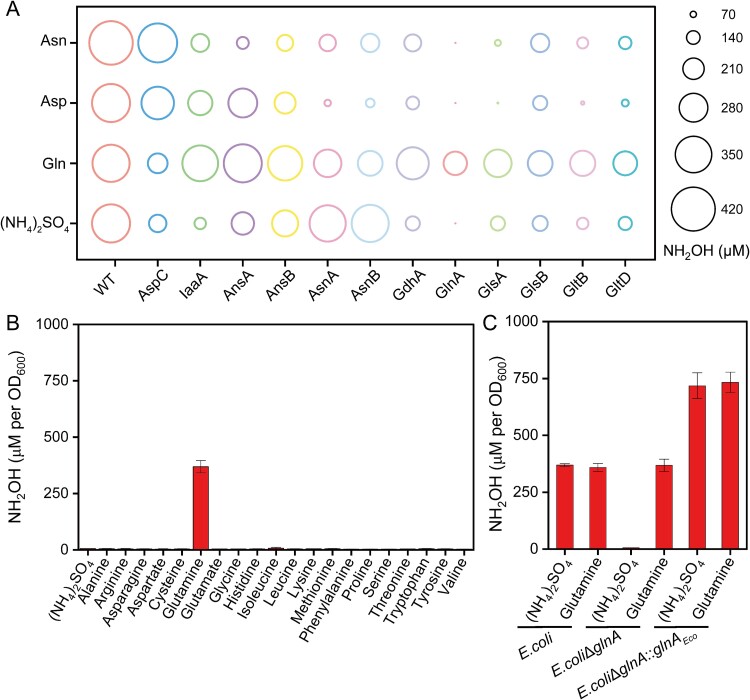
***E. Coli* cells harboring *dnfABC* require *glnA* to oxidize ammonia and amino acids, except glutamine.** (A) the accumulation of hydroxylamine in *E. Coli* mutant strains harboring *dnfABC* cultured with ammonia, glutamine, asparagine or aspartate. (B) the accumulations of hydroxylamine in *E. coli*Δ*glnA* harboring *dnfABC* with various nitrogen sources. (C) the accumulations of hydroxylamine in *E. Coli*, *E. coli*Δ*glnA*, and *E. coli*Δ*glnA*::*glnA* harboring *dnfABC* cultured with ammonia or glutamine. The data are represented as the mean ± s.d. of biological triplicates.

The deletions of individual genes had diverse effects on the ability to oxidize ammonia and amino acids (Fig. 3A, Supplementary Fig. 4B). The deletion of *glnA*, as opposed to other genes, completely eliminated the ability to oxidize ammonia, aspartate, asparagine, and the other amino acids, whereas still maintained the ability to oxidize glutamine with the hydroxylamine accumulation of 232.4 ± 3.4 μM (65% of WT) (Fig. 3A and 3B). The complementary strain (*E. coli*Δ*glnA*::*glnA_Eco_*-*dnfABC*) restored the ability to oxidize ammonia (Fig. 3C). The higher hydroxylamine accumulation of the complementary strain, compared to the wildtype *E. coli* strain, was presumed to be related to the higher *glnA* expression in the plasmid vector than in the chromosome. Among the tested genes, *glnA* was the only gene that abolished the ammonia oxidation ability. Meanwhile, the deletion of any tested gene did not eliminate the ability to oxidize glutamine (Fig. 3A). Considered that GlnA plays an essential role in the ammonia assimilation and nitrogen metabolism, we propose that GlnA could catalyze the conversion of ammonia, that were transported into cells from outsides or released from other amino acids inside cells, into glutamine, followed by subsequent oxidation to hydroxylamine via DnfABC. It was also noticed that the deletion of some genes almost abolished or substantially reduced the hydroxylamine production based on some amino acids, such as the *asnA*, *asnB*, *glsA*, *gltB*, and *gltD* mutants cultured with aspartate and the *glsA* mutant cultured with asparagine. We thought those mutants weakened bacterial ability to convert those amino acids to ammonia inside cells and then reduced the hydroxylamine production.

### Knockout of *glnA* in *A. faecalis* JQ135 resulted in the loss of heterotrophic ammonia oxidation ability but kept the ability to oxidize glutamine

To assess the role of *glnA* in *Alcaligenes*, we constructed the JQ135 cell with the *glnA* gene deletion (JQ135Δ*glnA*) and then tested its ammonia and amino acid oxidation ability in both growth-dependent and -independent assays. In the growth-dependent assays, it was challenging to evaluate the role of *glnA* in ammonia oxidation because JQ135Δ*glnA* was unable to grow with ammonia as the sole nitrogen source. In contrast, JQ135Δ*glnA* was able to grow using glutamine as the sole nitrogen source, and produced 129.6 ± 6.7 μM hydroxylamine, although with a prolonged lag phase and slower growth compared to JQ135 (Supplementary Fig. 5).

**Figure 4:**
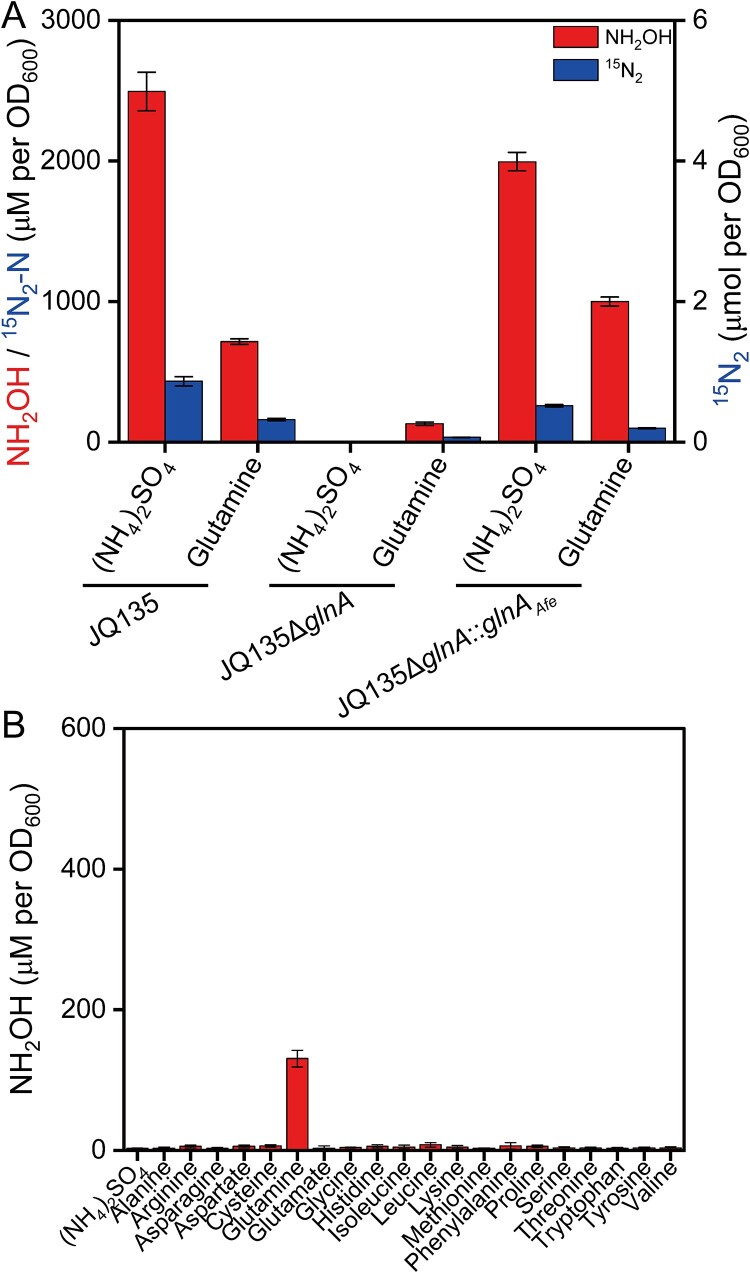
**The *glnA* gene is essential for *A. faecalis* JQ135 to oxidize ammonia.** (A) the accumulations of hydroxylamine and nitrogen gas of JQ135, JQ135Δ*glnA* and JQ135Δ*glnA*::*glnA_Afe_* cells cultured with ^15^N-labelled ammonia or ^15^N-labelled glutamine in whole cell transformation assays. ^15^N_2_-N stands for the concentration of ammonia-N that has been converted to ^15^N_2_. (B) the hydroxylamine accumulation of JQ135Δ*glnA* cultured with various nitrogen sources in whole cell transformation assays. The data are represented as the mean ± s.d. of biological triplicates.

**Figure 5:**
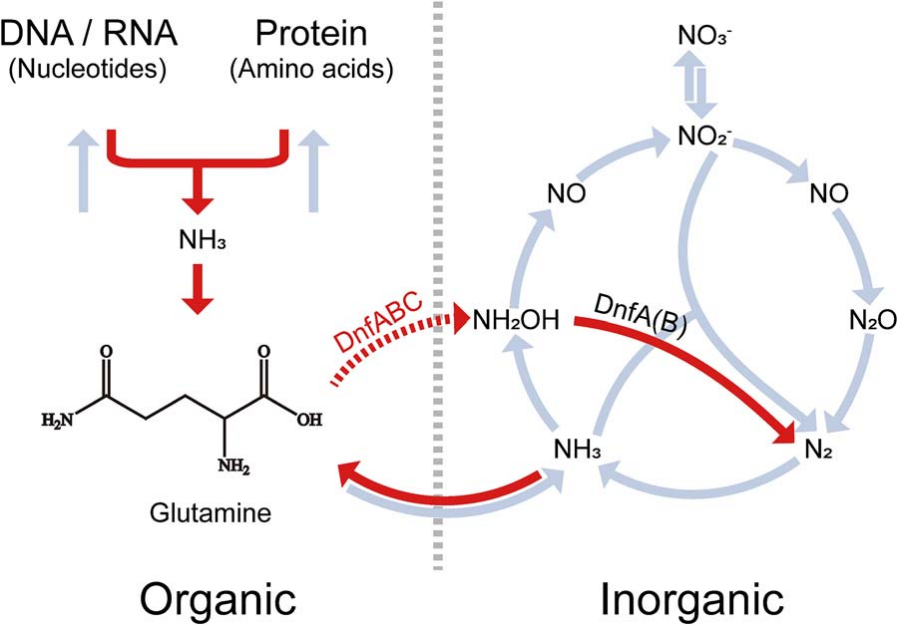
**The proposed dirammox pathway by *A. faecalis* JQ135.** The interconversion of DNA/RNA/proteins and glutamine are shown in left panel and the nitrogen cycle in right. The proposed dirammox pathway includes the conversion of ammonia to glutamine, then to NH_2_OH, and finally to N_2_. The conversion of glutamine to hydroxylamine (the dotted arrow) lacks the direct evidence and its mechanism remains unclear.

JQ135Δ*glnA* cells were cultured, harvested and then incubated with media containing ^15^N-isotype labelled ammonia and amide-^15^N-isotype labelled glutamine for 90 min in a sealed bottle filled with 80% He and 20% O_2_ to conduct a whole cell transformation assay (growth-independent). No hydroxylamine accumulation or ^15^N_2_ production was detected when JQ135Δ*glnA* cells were incubated with ^15^N-isotype labeled ammonia (Fig. 4A). In contrast, JQ135 and the complementary strain JQ135Δ*glnA*::*glnA_Afe_* showed hydroxylamine accumulations of 2495.6 ± 136.7 μM (9.98 ± 0.55 μmol) and 1995.6 ± 65.5 μM (7.98 ± 0.26 μmol), respectively, along with 0.86 ± 0.06 μmol and 0.52 ± 0.02 μmol ^15^N_2_ (Fig. 4A). The stoichiometries of hydroxylamine and N_2_-N accumulation by JQ135 and the complementary strain JQ135Δ*glnA*::*glnA_Afe_* were 5.8: 1 and 7.7: 1, respectively. When incubated with media containing 10 mM amide-^15^N-isotype labelled glutamine, JQ135, JQ135Δ*glnA*, and JQ135Δ*glnA*::*glnA_Afe_* produced 715.2 ± 20.4 μM (2.86 ± 0.08 μmol), 130.4 ± 12.1 μM (0.52 ± 0.05 μmol), and 1000.0 ± 31.1 μM (4.00 ± 0.12 μmol) of hydroxylamine and 0.32 ± 0.02 μmol, 0.07 ± 0.005 μmol, and 0.20 ± 0.01 μmol of ^15^N_2_ (Fig. 4A). The stoichiometries of hydroxylamine and N_2_-N accumulation by JQ135, JQ135Δ*glnA*, and JQ135Δ*glnA*::*glnA_Afe_* were 4.5: 1, 4.0: 1, and 10.0: 1, respectively. These results indicate that the deletion of *glnA* abolished the ammonia oxidation ability and somehow weakened the glutamine oxidation ability instead of completely abolishing it. To assess the role of *glnA* in the oxidation of other amino acids, JQ135Δ*glnA* cells were incubated with media containing various amino acids for 90 min to conduct a whole cell transformation assay (growth-independent). It was observed that the other amino acids could not be oxidized by the cells lacking *glnA*, indicating the role of GlnA in the amino acid oxidation (Fig. 4B). Taken together, *glnA* is necessary for JQ135 to oxidize ammonia and most amino acids except glutamine. The DnfA/B/C multienzyme system has been reported to could convert glutamine to N_2_, yet there was no evidence to support DnfA directly oxidizing glutamine [37]. Actually, DnfA did not interact with glutamine but with hydroxylamine [37]. DnfC could hydrolyze glutamine and L-glutamic acid γ-hydroxamate (L-GlnγHXM), a common characteristic of amidotransferases. We thus propose a hypothesis that ammonia and amino acids are first converted into glutamine by GlnA along with some deaminases, then to some unidentified intermediate by DnfC, and oxidized to hydroxylamine and finally to dinitrogen gas by DnfAB (Fig. 5).

## Discussion

Heterotrophic nitrification has been defined more broadly than the strict definitions of nitrification and anammox. Ammonia has been considered as the primary substrate for most studied microorganisms and has received significant attention in research on heterotrophic nitrification. In contrast, some microorganisms have displayed a wider range of substrates, including hydroxylamine, pyruvic oxime, and organic nitrogen [12, 18–26]. It was proposed that some organic nitrogen compounds such as pyridine and quinoline were converted to ammonia, then oxidized to nitrite and/or nitrate and finally reduced to dinitrogen gas in *Shinella zoogloeoides* and *Pseudomonas* sp. [41, 42]. To assess the substrate range of *A. faecalis* JQ135, tests were conducted using various amino acids, revealing the ability to oxidize different amino acids to hydroxylamine, regardless of whether the assays were growth-dependent or -independent. The deletion of *dnfA* gene, which abolished the organic nitrogen oxidation ability, suggested that cells recruit *dnfABC* to oxidize organic nitrogen, similar to the oxidation of ammonia (Supplementary Fig. 2). This was further supported by *E. coli* cells harboring *dnfABC*, which also exhibited the ability to oxidize amino acids, confirming the role of *dnfABC* in organic nitrogen oxidation. These findings suggest *A. faecalis* possesses a broader substrate range than previously known, as it is capable of oxidizing both ammonia and organic nitrogen via gene cluster *dnfABC* that was proposed to mediate the oxidation of ammonia to dinitrogen gas (the dirammox pathway). This observation provides support for considering the use of *A. faecalis* to remove organic nitrogen in wastewater treatment. The *dnfABC* homologs have been found in strains across different bacterial genera [33], such as *Microvigula aerodenitrificans* BE2.4, *Pseudomonas cedrina* LMG23661, *Delftia lacustris* HQS1, *Burkholderia pyrrocinia* LWK2, *Xanthobacter* sp. R2A-8, *Verminephrobacter aporrectodeae* At4, *Andreprevotia* sp. IGB-42, *Jeongeupia chitinilytica* KCTC23701, and *Pokkaliibacter plantistimulans* L1E11 (Supplementary Fig. 6). Furthermore, it is believed that other heterotrophic nitrifiers, including those encoded *dnfABC* or not, may also possess broader substrate range than previously known. A comprehensive survey of the substrates of heterotrophic nitrifiers would provide valuable insights for investigating heterotrophic nitrification and expanding the potential applications of these microorganisms.

Currently, there is limited information available regarding the enzymes involved in heterotrophic nitrification, especially those involved in ammonia oxidation. Among the enzymes that have been characterized, only HAO, cytochrome P460, and pyruvic oxime dioxygenase have been well studied for their biochemical, genetic, and physiological properties [6, 25, 26, 43–46]. However, there is currently no biochemical evidence supporting that ammonia or the tested organic nitrogen compounds are direct substrates for these potential ammonia oxidizing enzymes in heterotrophic nitrifiers. In contrast, enzymes involved in the oxidation of hydroxylamine or pyruvic oximes, including HAO, P460, POD, and DnfA, have been experimentally demonstrated to directly act on hydroxylamine or pyruvic oxime [34, 45, 46]. Our assays indicated that the deletion of glutamine synthetase gene *glnA* abolished the ability of *A. faecalis* JQ135 and *E. coli* cells harboring *dnfABC* to oxidize ammonia and organic nitrogen, except glutamine. This finding excludes the possibility that ammonia and most of those tested amino acids are the direct substrates for DnfABC. Instead, it suggests that ammonia transported into cells from outsides or released from organic nitrogen inside cells were converted into glutamine and finally oxidized to hydroxylamine and dinitrogen gas by DnfABC (Fig. 5). Miao et al. observed that DnfABC converted glutamine to nitrogen gas in vitro and proposed that glutamine was oxidized to L-glutamic acid γ-hydroxamate (L-GlnγHXM) by DnfAB, hydrolyzed to hydroxylamine by DnfC and then oxidized to dinitrogen gas by DnfAB. Yet it lacked direct biochemical evidence for DnfA oxidizing glutamine [37]. Until now, we did not obtain direct evidence of DnfA oxidizing glutamine. We observed that DnfA interacted with hydroxylamine but not with glutamine using ITC and DSF assays [34]. Miao et al. also reported that DnfA did not interact with glutamine but with hydroxylamine [37]. We therefore concluded that DnfA oxidizes some unidentified intermediate rather than glutamine. DnfC was predicted to be a potential glutamine amidotransferase and essential for ammonia oxidation. The reported hydrolysis activity of glutamine and L-glutamic acid γ-hydroxamate (L-GlnγHXM) by DnfC was possibly an intrinsic feature of glutamine amidotransferases. Additionally, the expression levels of DnfC varied significantly in JQ135 cells cultured with glutamine, asparagine, and aspartate, yet the hydroxylamine accumulations of those cells were comparable to each other. This observation contradicts the hypothesis proposed by Miao et al., which suggested that a high concentration of DnfC would inhibit hydroxylamine production. Some *Alcaligenes* members encoding *dnfABC* were observed not to oxidize ammonia to hydroxylamine [31], suggesting DnfABC enzymes and substrate glutamine were necessary but insufficient. Based on these observations, it can be inferred that glutamine is not a direct substrate of DnfA, emphasizing the intricate biochemical and physiological characteristics involved in heterotrophic nitrification. Although direct evidence to confirm or exclude glutamine as the direct substrate of DnfA was not provided, it is plausible that glutamine undergoes conversion into an unidentified organic nitrogen compound before oxidation occurs. Recently, Lenferink et al. observed that addition of 2.5 mM ^14^NH_4_Cl, 500 μM Na^14^NO_2_, and then 250 μM ^15^NH_2_OH (added 1.5 h after the addition of ^14^NH_4_Cl and ^14^NaNO_2_) generated ~10 μM ^30^N_2_-N (4% of ^15^NH_2_OH-N) after 4–8 h in sterile medium or medium containing heat-killed *Alcaligenes* cells, and only 1–2 μM ^30^N_2_-N in medium containing *Alcaligenes* cells, with conversion of hydroxylamine to N_2_ being considered an abiotic process [38]. ^14^NH_2_OH was produced at 1.5 h in media containing living cells in these assays and hydroxylamine concentrations (^14^NH_2_OH and ^15^NH_2_OH) were maintained at 500 μM from 4 to 8 h after the addition of 250 μM ^15^NH_2_OH [38]. The added ^15^NH_2_OH would react with ^14^NH_2_OH produced from ^14^NH_4_^+^ to generate ^29^N_2_, which complicated the interpretation. We believe Lenferink observed an abiotic conversion of hydroxylamine as with our biochemical assays, but this did not conclusively demonstrate a lack of biotic oxidation of hydroxylamine to N_2_ by DnfA that might dominate the conversion of hydroxylamine to N_2_ under some conditions.

In the growth-dependent assays, it was observed that the expression of DnfA/DnfC was not consistently correlated with hydroxylamine accumulation during the oxidation of ammonia and amino acids by JQ135, indicating that the presence of a regulatory mechanism independent of gene expression that influenced hydroxylamine production. The assimilation of ammonia into glutamine by GlnA could potentially be one of the processes involved in regulating ammonia oxidation. Furthermore, we noticed that the oxidation of ammonia in JQ135 was significantly faster than that of glutamine in the growth-independent assay despite the established role of glutamine as an essential intermediate for ammonia oxidation, whereas the oxidation velocities of ammonia and glutamine were found to be similar in *E. coli* cells harboring *dnfABC*. The faster oxidation rate observed for ammonia suggests the involvement of unidentified rate-limiting steps specific to ammonia oxidation pathway, which differ from those associated with glutamine. The different ammonia and glutamine transporters might be one rate-limited step. Also, the synthesis of the proposed unidentified intermediate might be another rate-limiting step. We believe the presence of ammonia, rather than glutamine, likely enhances the synthesis of those rate-limiting intermediates.

The assimilation of ammonia requires energy, which could account for one of the reasons why heterotrophic nitrification necessitates the presence of additional organic matter. Furthermore, the absence of autotrophic *amoA* genes in the genomes of identified heterotrophic nitrifiers supports ammonia assimilation prior to oxidation. The reported heterotrophic *amoA* gene in *Pseudomonas* sp. has been discredited as associated with heterotrophic nitrification due to its sequence containing fragments from two neighboring genes [33]. Furthermore, the fact that the deletion of its homolog had no impact on the ammonia oxidation ability of *A. faecalis* JQ135 lends further support to this conclusion [33, 47]. Therefore, we propose a hypothesis that ammonia-oxidizing heterotrophic nitrifiers assimilate ammonia and subsequently oxidize unidentified amines to hydroxylamine and finally to dinitrogen gas, nitrite or nitrate, just like *A. faecalis* JQ135 and the artificially engineered heterotrophic nitrifier *E. coli* cells did. The other two enzymes involved in ammonia assimilation were not necessary for ammonia oxidation, i.e. GDH that catalyzes the reversible formation of 2-oxoglutarate to glutamate and that GOGAT catalyzes the formation of two glutamate molecules from 2-oxoglutarate and glutamine. Additional challenging and direct evidence is required to understand the intricate mechanism of ammonia oxidation fully. The single gene knockout approach used here is not enough for identifying those genes involved in dirammox. A mutagenesis library should be a much more appropriate choice to reveal all the genes involved in the dirammox pathway. Multi-omics assays would highlight those genes, proteins, and metabolites important for dirammox. Further, the identification of the DnfR and/or DnfC ligands via co-purification would provide us a direct clue for the substrate of DnfABC. Nonetheless, we remain committed to uncovering the ammonia oxidation pathway in the following study.

Converting glutamine to hydroxylamine by DnfABC shunts the organic nitrogen metabolism to the inorganic nitrogen one. Until now, the precise role of heterotrophic nitrification in the nitrogen cycle remains unclear, apart from its speculated involvement in specific niches. For example, heterotrophic nitrification has been proposed to respond to produce N_2_O in acid soils where the autotrophic nitrifiers were inhibited by low pH and could not produce N_2_O [48]. Previously, heterotrophic nitrification has been proposed to occur as an inorganic pathway or an organic pathway [49]. Some studies have also shown the effect of organic nitrogen on heterotrophic nitrification and the production of N_2_O during heterotrophic nitrification [50]. In this study, we present compelling biological evidence to establish a direct link between organic nitrogen metabolism and the nitrogen biogeochemical cycle with glutamine identified as the intermediary compound in this linkage. Our findings have identified an additional pathway connects organic and inorganic nitrogen metabolism, which was previously though to primarily involve ammonization and ammonia assimilation. Given the prevalence of organic nitrogen and the wide distribution of heterotrophic nitrifiers *Alcaligenes* in the environment [31], it is imperative that we pay closer attention to the shunt from central nitrogen metabolism to inorganic nitrogen to fully assess the role of dirammox pathway in the nitrogen cycle.

The adaptive advantage is an obvious question for understanding dirammox. In contrast to the obvious role in energy generation of nitrification and anammox, the advantage of dirammox remains mystery. It has been speculated that bacteria could balance electron transfer and neutralize the toxicity of high concentrations of ammonia via dirammox [32]. Bacteria could also inhibit the growth of other strains via hydroxylamine and then adapt to environments with competitors [51, 52]. We found that hydroxylamine inhibited the growth of JQ135 and DnfA deletion mutant (JQ135Δ*dnfA*) with IC_50_ values of 2.5 ± 0.2 mM and 2.5 ± 0.3 mM, respectively (Supplementary Fig. 7). The IC_50_ values are higher than the hydroxylamine accumulation concentrations during growth, suggesting that JQ135 could tolerate the hydroxylamine itself produced via some unknown mechanisms. The sequential conversion of hydroxylamine to N_2_ could prevent accumulation of high concentration hydroxylamine. The oxidation of ammonia to hydroxylamine, using glutamine as an intermediate, could be easily regulated by the bacterial carbon/nitrogen ratio, preventing the consumption of excess nitrogen sources beyond bacterial growth.

In summary, our study revealed that heterotrophic nitrifier *A. faecalis* JQ135 oxidized ammonia and amines by first converting them to glutamine. We therefore hypothesized most heterotrophic nitrifiers that did not encode any AMO homologs might oxidize ammonia after it has been assimilation. This hypothesis challenges the paradigm of heterotrophic nitrification. The conversion from glutamine to hydroxylamine by dirammox enzymes DnfABC introduces an additional pathway connecting organic and inorganic nitrogen metabolisms, supplementing the existing ammonization and ammonia assimilation routes (Fig. 5). Consequently, further investigation is warranted to explore the role of this shunt in the nitrogen cycle.

## Supplementary Material

Supplementary_information-240907_wrae174

Supplementary_Table_1_wrae174

Supplementary_Table_2_wrae174

## Data Availability

All data generated or analyzed during this study are included in this published article and its supplementary information files.
