# Supplementary material for: Heterotrophic nitrification by Alcaligenes faecalis links organic and inorganic nitrogen metabolism
Source: ISME J. 2024 Sep 10;18(1):wrae174. doi: 10.1093/ismejo/wrae174 (PMC11440038; doi:10.1093/ismejo/wrae174)
Supplement: Supplementary_information-240907_wrae174 [file supplementary_information-240907_wrae174.docx]

**Supplementary Information**


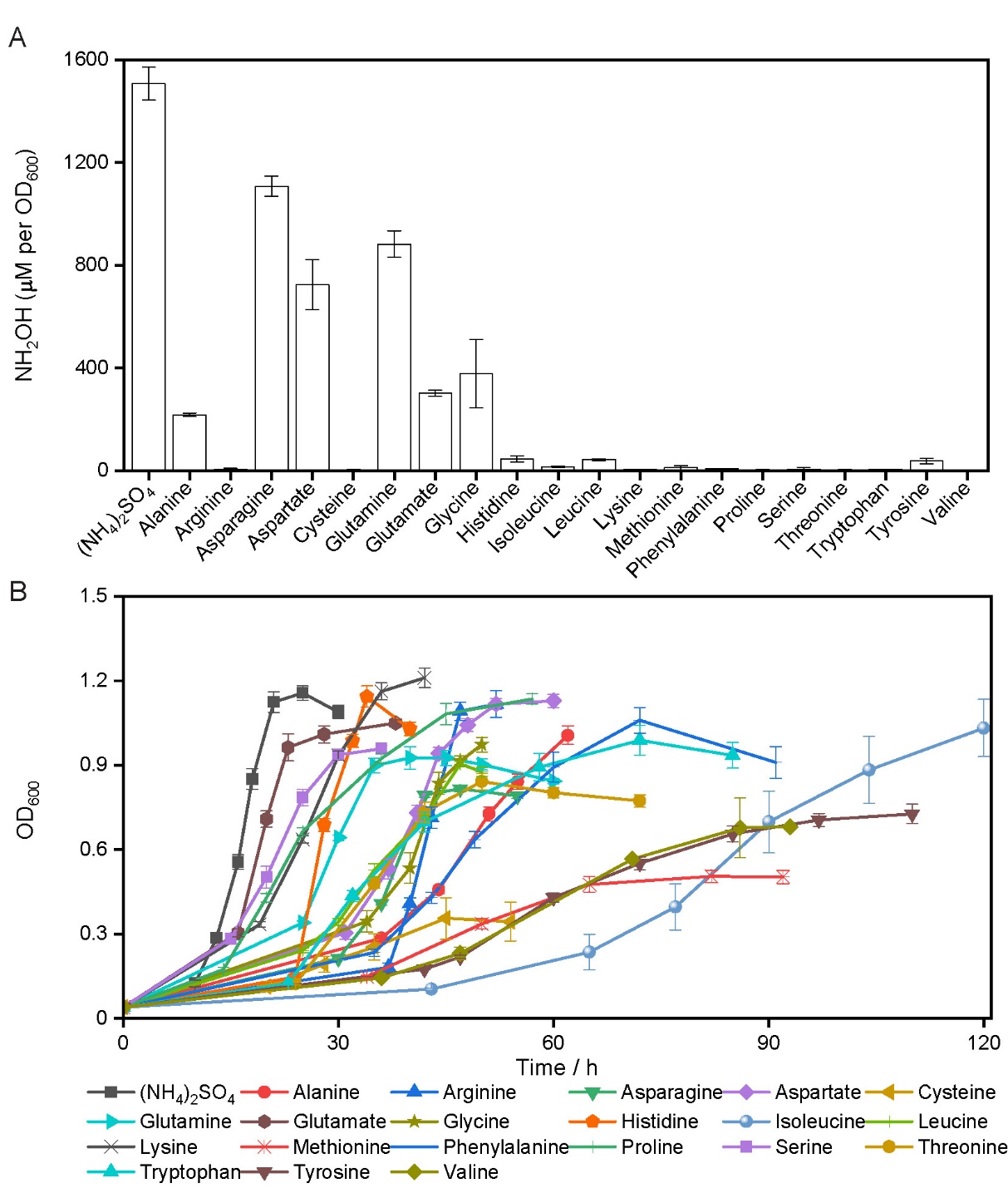


**Supplementary Figure 1. *Alcaligenes faecalis* JQ135 cells oxidize amino acids besides ammonia in growth-dependent assays.** The observed maximum accumulations of hydroxylamine (A) and cell growth curves (B) of *Alcaligenes faecalis* JQ135 cultured with various nitrogen sources. The data is represented as the mean ± s.d. of biological triplicates.


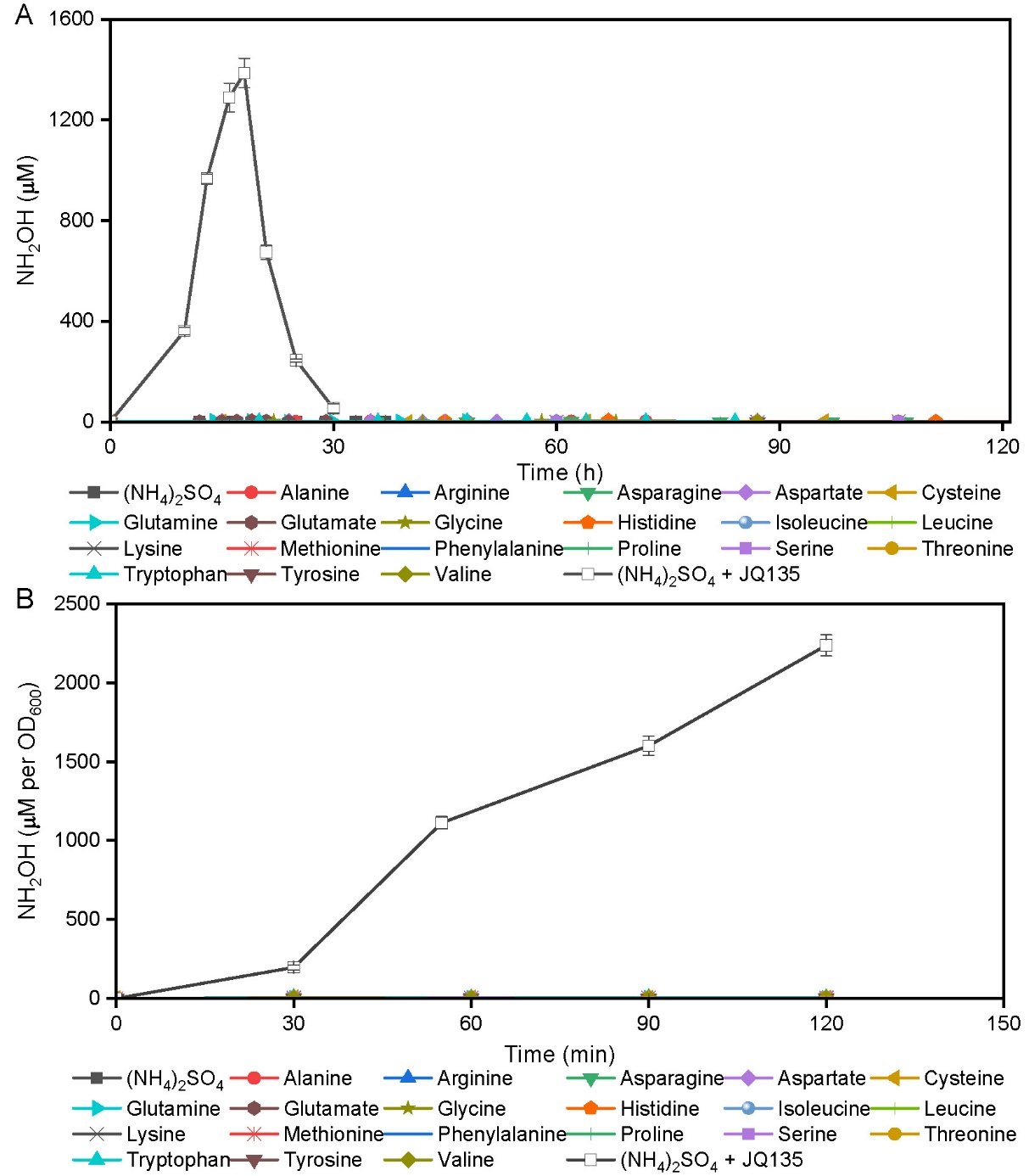


**Supplementary Figure 2. *Alcaligenes faecalis* JQ135 cells oxidize amino acids to hydroxylamine through the dirammox pathway.** Hydroxylamine accumulation curves of *Alcaligenes faecalis* JQ135Δ*dnfA* cultured with various nitrogen sources in growth-dependent assays (A) and whole cell transformation assays (B). The data is represented as the mean ± s.d. of biological triplicates.


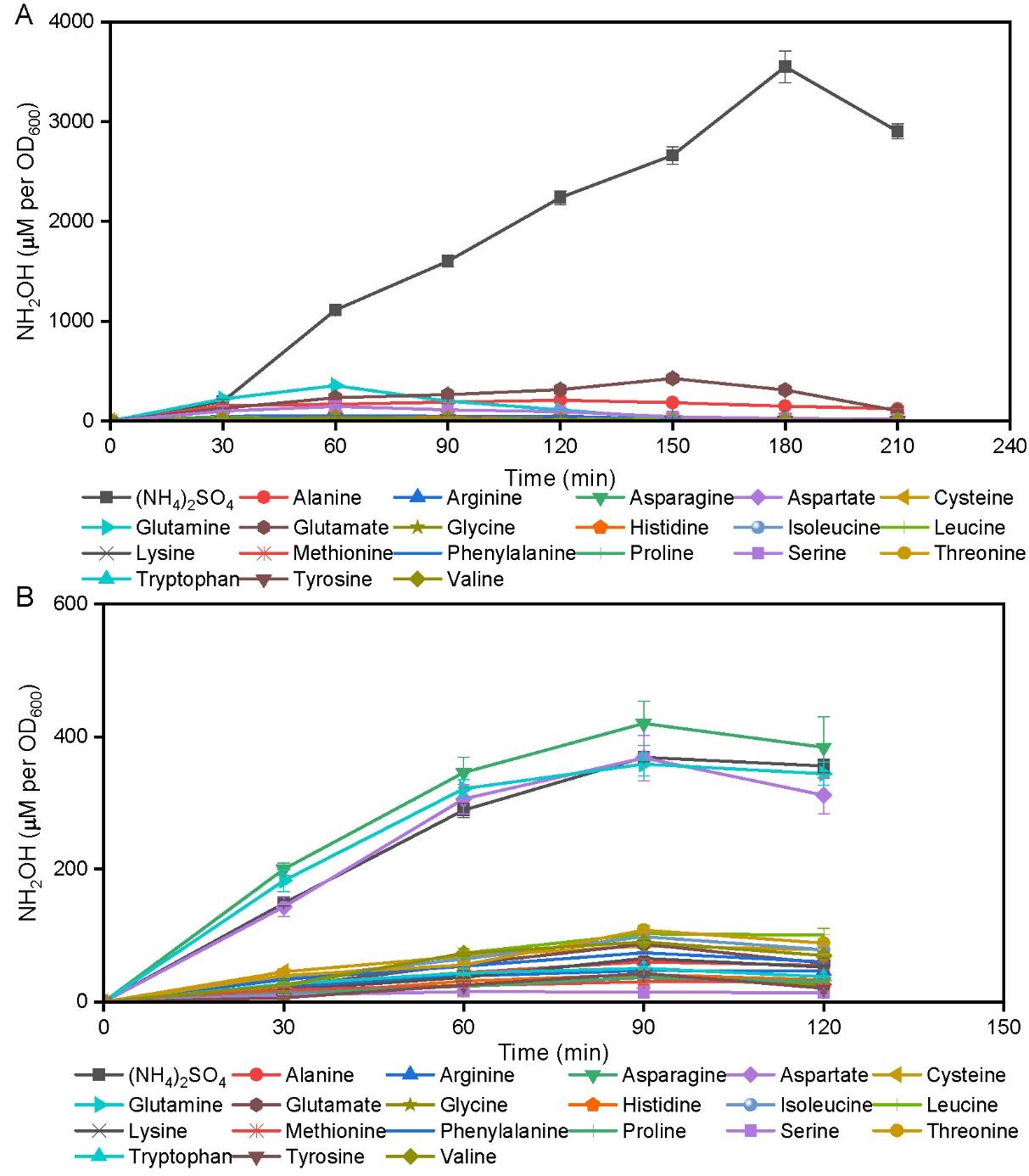


**Supplementary Figure 3. *Alcaligenes* *faecalis* JQ135 and *E*. *coli* cells harboring *dnfABC* oxidize amino acids besides ammonia in whole cell transformation assays.** Hydroxylamine accumulation curves of *Alcaligenes faecalis* JQ135 (A) and *E*. *coli* cells harboring *dnfABC* (B) cultured with various nitrogen sources in whole cell transformation assays. The data is represented as the mean ± s.d. of biological triplicates.


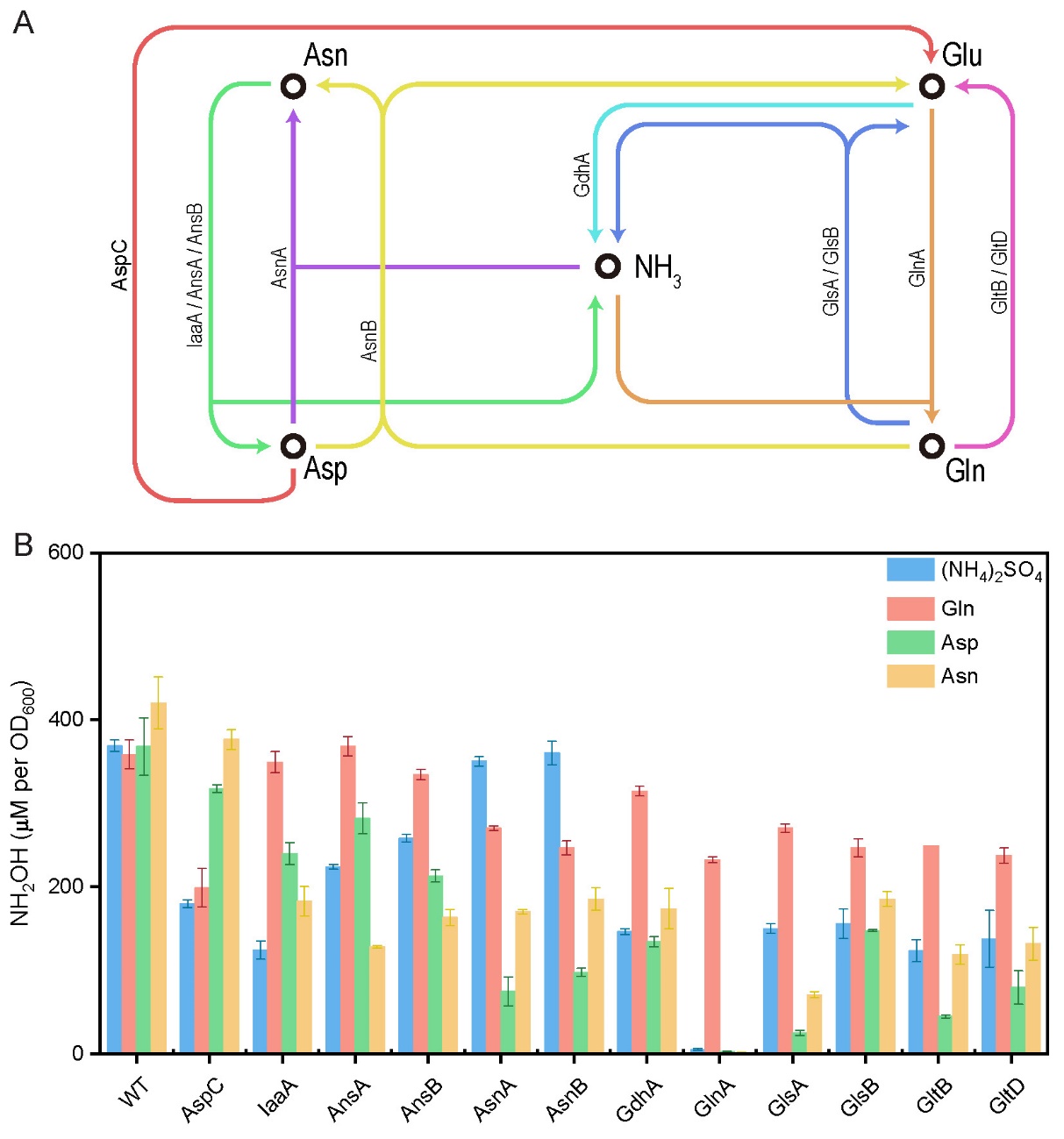


**Supplementary Figure 4. The key genes involved in the interconversion of ammonia, glutamine, glutamate, asparagine and aspartate as well as the reactions they participate in in *E*. *coli*.**

(A) The roles of genes involved in nitrogen metabolism. AspC converts aspartate to glutamate, AsnA converts ammonia and aspartate to asparagine, IaaA, AnsA, and AnsB convert asparagine to ammonia and aspartate, GlnA converts ammonia and glutamate to glutamine, GltBD converts glutamine and 2-oxoglutarate to glutamate, GlsA and GlsB convert glutamine to ammonia and glutamate, GdhA converts glutamate to ammonia and 2-oxoglutarate, and AsnB converts aspartate and glutamine to asparagine and glutamate. (B) The maximum accumulations of hydroxylamine in *E*. *coli* mutant strains harboring *dnfABC* cultured with ammonia or amino acids. The data is represented as the mean ± s.d. of biological triplicates.


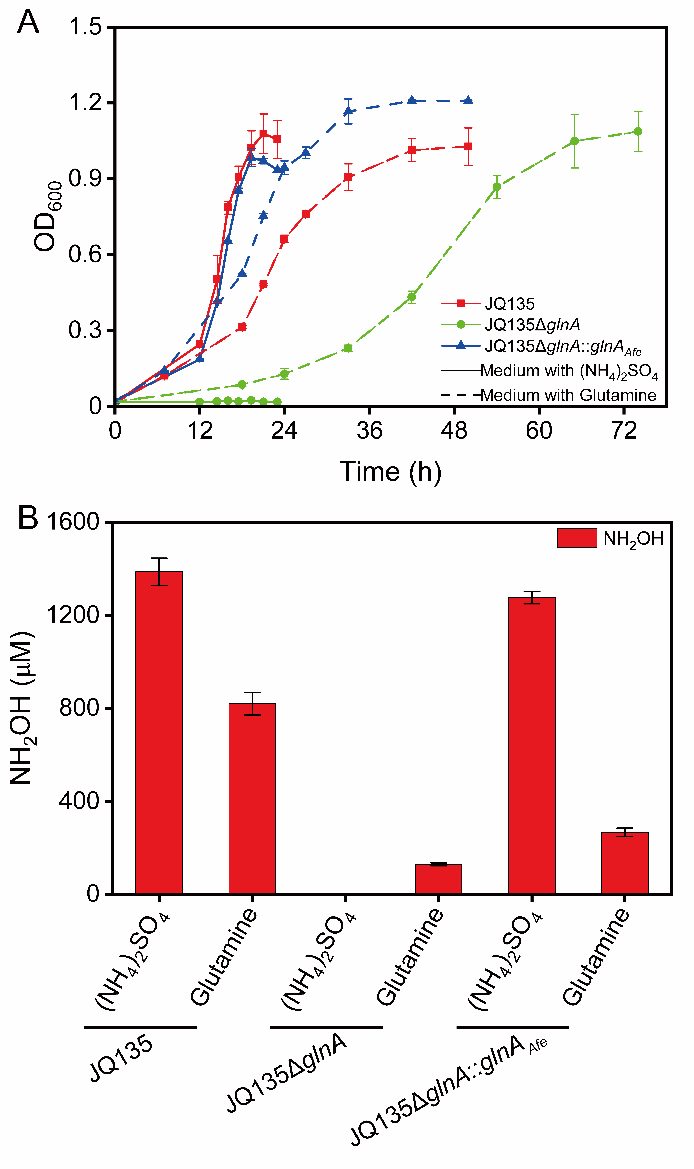


**Supplementary Figure 5. The *glnA* gene is essential for *Alcaligenes* *faecalis* JQ135 to oxidize ammonia but not glutamine in the growth-dependent assays.**

The cell growth curves (A) and hydroxylamine accumulations (B) of JQ135, JQ135Δ*glnA* and JQ135Δ*glnA*::*glnA_Afe_* cultured with ammonia or glutamine in growth-dependent assays. The data is represented as the mean ± s.d. of biological triplicates.


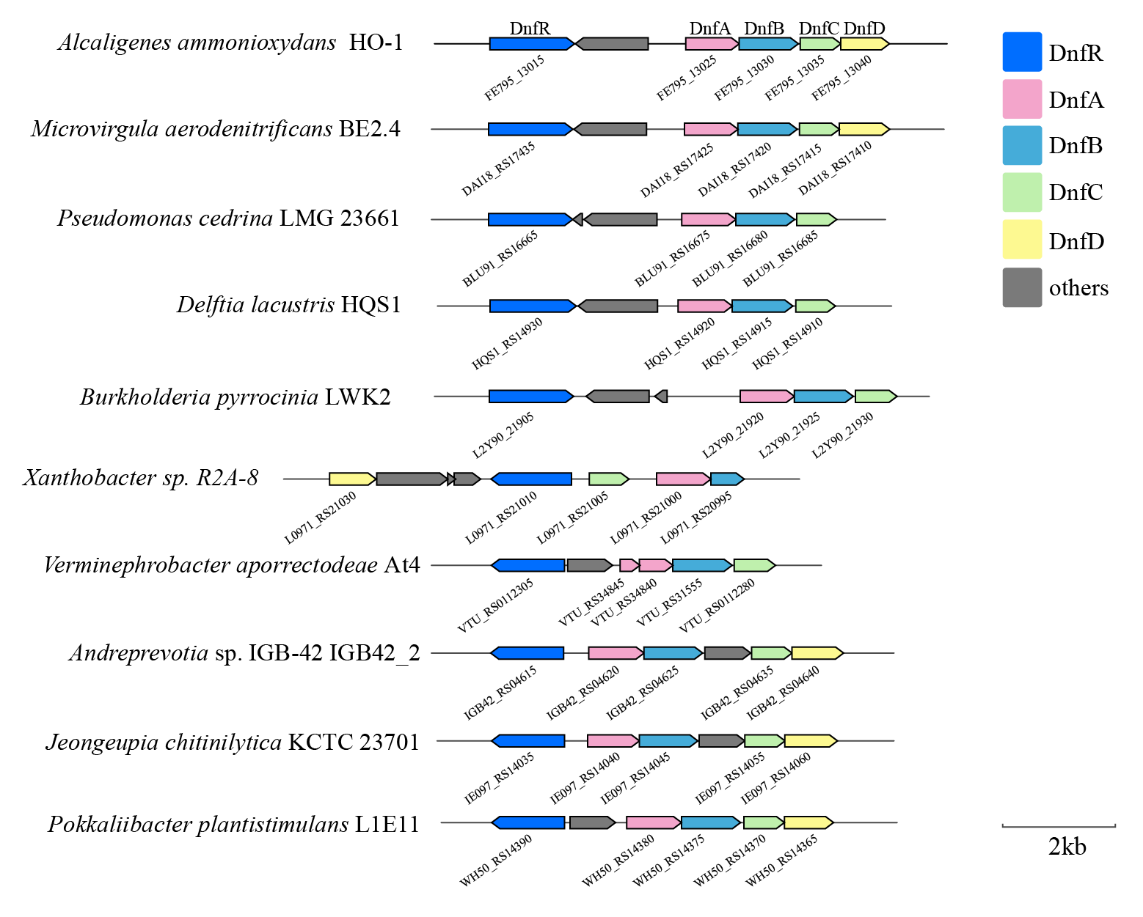


**Supplementary Figure 6. The arrangements of gene cluster *dnfRABC* in strains from different genera.**


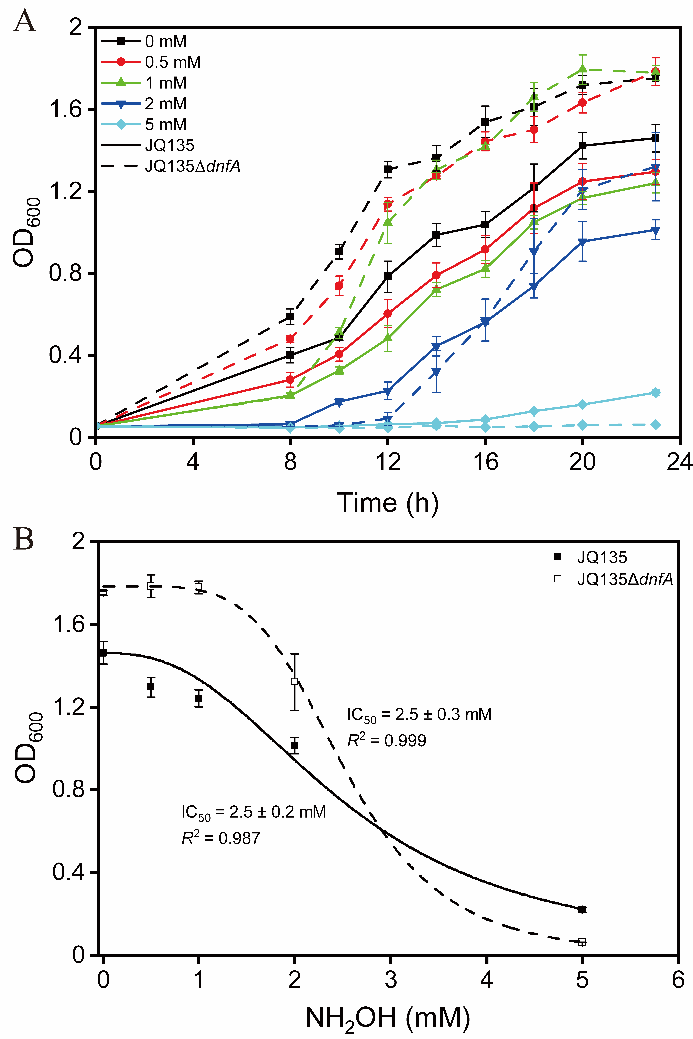


**Supplementary Figure 7. The inhibition of hydroxylamine against *Alcaligenes* *faecalis* JQ135 and JQ135Δ*dnfA*.**

Bacterial growth curves (A) and OD_600_ at 23 hours (B) for strains JQ135 and JQ135Δ*dnfA* cultured in LB medium with varying hydroxylamine concentrations. The data was fitted in a logistic equation. The data is represented as the mean ± s.d. of biological triplicates.

**Supplementary Table 1**. **Strains and plasmids used in this study.**

| Strains or plasmids | | Description | Source |
| --- | --- | --- | --- |
| Strains |  | |  |
| *A*. *faecalis* JQ135 | Str^r^; wild type | | [1] |
| HB101 (pRK2013) | Help strain for biparental mating | | [1] |
| DH5α | Cloning host | | Lab stock |
| *E.coli* BW25113 | Wild type, protein expression host | | [2] |
| *E.coli* BW25113 Δ*aspC* | Km^r^; *aspC* deletion mutant of *E.coli* BW25113 | | [2] |
| *E.coli* BW25113 Δ*iaaA* | Km^r^; *iaaA* deletion mutant of *E.coli* BW25113 | | [2] |
| *E.coli* BW25113 Δ*ansA* | Km^r^; *ansA* deletion mutant of *E.coli* BW25113 | | [2] |
| *E.coli* BW25113 Δ*ansB* | Km^r^; *ansB* deletion mutant of *E.coli* BW25113 | | [2] |
| *E.coli* BW25113 Δ*asnA* | Km^r^; *asnA* deletion mutant of *E.coli* BW25113 | | [2] |
| *E.coli* BW25113 Δ*asnB* | Km^r^; *asnB* deletion mutant of *E.coli* BW25113 | | [2] |
| *E.coli* BW25113 Δ*gdhA* | Km^r^; *gdhA* deletion mutant of *E.coli* BW25113 | | [2] |
| *E.coli* BW25113 Δ*glnA* | Km^r^; *glnA* deletion mutant of *E.coli* BW25113 | | [2] |
| *E.coli* BW25113 Δ*glsA* | Km^r^; *glsA* deletion mutant of *E.coli* BW25113 | | [2] |
| *E.coli* BW25113 Δ*glsB* | Km^r^; *glsB* deletion mutant of *E.coli* BW25113 | | [2] |
| *E.coli* BW25113 Δ*gltB* | Km^r^; *gltB* deletion mutant of *E.coli* BW25113 | | [2] |
| *E.coli* BW25113 Δ*gltD* | Km^r^; *gltD* deletion mutant of *E.coli* BW25113 | | [2] |
| Plasmids |  | |  |
| pBAD | Gene expression plasmid | | Lab stock |
| pBAD-*dnfABC* | pBAD carrying *dnfABC* | | This study |
| pJQ200SK | Gm^r^; suicide plasmid | | [1] |
| pJQ200SK-Δ*glnA* | Gm^r^; *glnA* gene deletion plasmid based on pJQ200SK | | This study |
| pBBR1MCS5 | Gm^r^; broad-host-range cloning plasmid | | Lab stock |
| pBBR-*glnA_Eco_* | Gm^r^; pBBR1MCS5 harboring *glnA_Eco_* | | This study |
| pBBR-*glnA_Afe_* | Gmr; pBBR1MCS5 harboring *glnA_Afe_* | | This study |
| JQ135Δ*glnA* | Str^r^, Km^r^; *glnA* deletion mutant of JQ135 | | This study |
| JQ135Δ*glnA*::*glnA_Afe_* | Str^r^, Km^r^, Gm^r^; JQ135Δ*glnA* containing pBBR-*glnA_Afe_* | | This study |
| *E.coli* BW25113 Δ*glnA*::*glnA_Eco_* | Km^r^, Gm^r^; *E.coli* BW25113 *ΔglnA* containing pBBR-*glnA_Eco_* | | This study |

**Supplementary Table 2**. **Primers used in this study.**

| Primers | Sequence (5' to 3') | Description |
| --- | --- | --- |
| dnfABC-F | GTGTGCTAGCATGACAATCAAAAGCTACGAAAC | To construct plasmid pBAD-dnfABC |
| dnfABC-R | GTGTCTCGAGTCATGCAGCACAATCAGCG |  |
| koglnA-UF | ATTGGGTACCGGGCCCCCCCTCGAGAGCGCGTCAATCTGGACATC | To construct plasmid pJQ200SK-ΔglnA |
| koglnA-UR | GACACCAGACTCAAAGCGAT |  |
| koglnA-DF | CAACTGGAAATCGGCACTCG |  |
| koglnA-DR | CGGCCGCTCTAGAACTAGTGGATCCTCTCGCGTGGACAGATCGTC |  |
| glnA_Eco_-F | GTGTCTCGAGACATCCTCCGCAAACAAGTATTG | To construct plasmid pBBR-glnA*_Eco_* |
| glnA_Eco_-R | GTGTTCTAGATTAGACGCTGTAGTACAGCTCAAAC |  |
| glnA_Afe_-F | GTGCCTCGAGAAGGCTCCTGACATTTAATTGTTTATT | To construct plasmid pBBR-glnA_Afe_ |
| glnA_Afe_-R | GTGTGGATCCTTACAGGCCGTAGTACATGTCG |  |

**References**

1. Xu SQ, Qian XX, Jiang YH, Qin YL, Zhang FY, Zhang KY *et* *al*. Genetic foundations of direct ammonia oxidation (dirammox) to N_2_ and MocR-like transcriptional regulator DnfR in *Alcaligenes* *faecalis* strain JQ135. *Appl Environ Microbiol*. 2022;88:e0226121.

2. Baba T, Ara T, Hasegawa M, Takai Y, Okumura Y, Baba M *et al*. Construction of *Escherichia* *coli* K-12 in-frame, single-gene knockout mutants: the Keio collection. *Molecular Systems Biology*. 2006;2:2006-0008.
